# Your “out of the office” email is not fooling me

**DOI:** 10.1038/s44319-024-00145-2

**Published:** 2024-05-07

**Authors:** David R Smith

**Affiliations:** Western Ontario University, London, ON Canada

**Keywords:** History & Philosophy of Science, Methods & Resources

## Abstract

Don’t fool yourself to think that you are safe from emails just because you enabled the “Out of office” function. It is not an ‘Off’ button that grants you reprieve from daily chores.

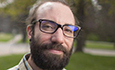

You checked in to your flight precisely 24 h before departure. The boarding passes are safely downloaded on your smartphone. The reliable carry-on bags are packed and sitting by the front door. No checked luggage: you are a pro. You have all the right cables for all the wrong places. Adapters galore. Your presentation is backed up on a thumb drive and Dropbox. You have some sneakers and shorts in case there’s time between talks to sneak out for a run. For 5 full days, you will be free from all those pesky domestic duties. Garbage and recycling? The reliable neighbor will take care of that. Picking up the kids, making dinner, and packing lunches? The supportive partner or grandparents will have to step up. You are on your way. Soon, you will be sitting in an airport café or lounge, eating roasted almonds, sipping soda water or chardonnay, chugging along on your laptop, and getting things done. But there is one last chore before you leave, perhaps the most important one. You must engage the “out of the office” email. No academic journey is complete without the assurance that a short, polite note will automatically respond to all incoming emails. You know what I mean, the one that says, in so many words: “Na-na na-na na-na. I am at a conference. I have a get-out-of-email-free card!”

But you are not fooling me. I know that the minute you sit in your taxi, the second your flight touches down, the moment you arrive at the conference hall… you will be refreshing your inbox. In fact, you’ll probably go out of your way to find the best Wi-Fi spot in the entire venue and watch the emails pour in. You will tell yourself: “It’s all good. No hard feelings. That little automatic response is taking care of everything.” But you will betray yourself. Someone will send an email that is just too irresistible to ignore. You will respond—maybe only a few words or an emoji—and the jig is up. The receiver will look in disbelief. How can this be? And only minutes after the automatic reply? The veil will dissolve. They will realize you are not trekking through a remote jungle in Borneo, climbing a glacier in the Andes, or kayaking to an uninhabited island in the Antarctic. You are sitting on a modestly comfortable hotel couch working through a to-do list in between talks. One could even argue that because you are sheltered from the mundane hustle and bustle of daily life, you are less busy than your at-home colleagues who are enduring dirty dishes, rush-hour traffic, and Costco.

I can hear the readers moaning: “Come on, Dave. You are going too far. Do not be such a cynic.” Maybe I got a chip on my shoulder. The truth is, I have been burned badly by the “out of office email,” and it was all my fault. About ten years ago, when I was a newly minted assistant professor, I was getting ready to leave for a conference in Spain. I said to myself, “Dude, go for it. You’ve finally reached the appropriate level of responsibility to employ the out-of-office email.” I savored the moment. With a hot coffee and biscuit by my side, I opened the settings tab of Apple Mail and drafted the most eloquent, gentle automatic response ever written. Turning it on was trickier than I expected, with various boxes to tick and untick, but eventually I clicked OK and felt an immediate sense of self-importance.

Moments later, my inbox began to ding relentlessly with incoming mail. “What is this,” I said, scratching my head. Ironically, I was receiving a barrage of “out of the office” emails from individuals I had not written to. Or had I? I quickly realized my mistake. Instead of selecting the option whereby all new incoming mail triggered an automatic response, I’d chosen the setting where all emails in my account, old and new, received the response. So what? Well, I’m one of those people who saves *all* my emails in hundreds of different subfolders. This meant thousands of people—students, colleagues, ornery editors, old cat sitters—received a letter notifying them that Prof. David Smith was on his way to Spain for important business, so do not bother emailing him back. That was the beginning and the end of my journey with the automatic reply. I swore never to go near it again. And I have stuck to my guns.

Over the last decade, I’ve had a gentleman’s agreement with the “out of the office” email. When possible, we avoid each other. But something happened a few weeks ago that pushed me over the edge, compelling me to write this essay. I was puttering away peacefully on a lecture when an email notification caught my attention. An old friend from another university was asking for a favor. After debating my options, I replied that I was happy to help. What should happen a few moments later? You guessed it: an automatic response from my friend. Looking closely, I saw that something was off. This was not an ordinary “out of the office” reply, it was an “in the office” automatic reply. I reread the email a few times: *Owing to many commitments, imminent important deadlines, and a backlog of unfinished tasks, I may be unable to respond to your email. FYI: I am unlikely to be able to fulfill any last-minute requests that require a lot of time or effort from me. Thank you for your patience*. I sat in my office chair, defeated. The automatic reply had won. My only condolence was that this person was at least not pretending to be miles away from an internet connection.

The truth is that so many of us are seeking that unattainable “off” button. A reprieve from the tiresome tasks, problems and requests that confront us each day. A way to say to the world, “Back off. Leave me alone.” I don’t blame anyone for trying to find refuge in an email setting, but maybe it’s only a band-aid for a much deeper issue. But I am laying down my arms. I promise not to judge you if you keep turning on that automatic reply. Your secret is safe with me. But you better believe that any letters going to dsmit242@uwo.ca will receive a prompt, customized, intentional, and heartfelt reply.

### Supplementary information

Peer Review File

