# Supplementary material for: Your “out of the office” email is not fooling me
Source: EMBO Rep. 2024 May 7;25(6):2511–2. doi: 10.1038/s44319-024-00145-2 (PMC11169599; doi:10.1038/s44319-024-00145-2)
Supplement: Supplementary file 1 — Peer Review File [file 44319_2024_145_MOESM1_ESM.pdf]

EMBO reports

Your "out of the office" email isn't fooling me.

David Smith

Corresponding author(s): David Smith (dsmit242@uwo.ca)

**Review Timeline:**	Submission Date:	12th Apr 24
	Accepted:	17th Apr 24

Editor: Holger Breithaupt

Transaction Report: This article type is not peer reviewed.
